# Supplementary material for: Lessons Learned from Translating Genome Sequencing to Clinical Routine: Understanding the Accuracy of a Diagnostic Pipeline
Source: Genes (Basel). 2024 Jan 22;15(1):136. doi: 10.3390/genes15010136 (PMC10815474; doi:10.3390/genes15010136)

SUPPLEMENTARY TABLES AND FIGURES

Supplementary Table S1: Quality parameters

Data analytic step	Metric	Details
Raw data (FASTQ)	read count	Total number of reads (one cluster in a paired-end experiment generates two reads).
	read length	Raw read length of a single read before trimming. Comma-separated list of lengths if several.
	Q20 read percentage	The percentage of reads with a mean base quality score greater than Q20.
	Q30 base percentage	The percentage of bases with a minimum quality score of Q30.
	no base call percentage	The percentage of bases without base call (N).
	gc content percentage	The percentage of bases that are called to be G or C.
	bases sequenced (MB)	Bases sequenced in total (in megabases).
Mapping (BAM file)	trimmed base percentage	Percentage of bases that were trimmed during to adapter or quality trimming.
	mapped read percentage	Percentage of reads that could be mapped to the reference genome.
	on-target read percentage	Percentage of reads that could be mapped to the target region.
	properly-paired read percentage	Percentage of properly paired reads (for paired-end reads only).
	insert size	Average insert size (for paired-end reads only).
	duplicate read percentage	Percentage of reads removed because they were duplicates (PCR, optical, etc).
	target region read depth	Average sequencing depth in target region.
	target region 20x percentage	Percentage of the target region that is covered at least 20-fold.
	bases usable (MB)	Bases sequenced that are usable for variant calling (in megabases).
	SNV allele frequency deviation	Percentage of common SNPs that deviate from the expected allele frequency (i.e. 0.0, 0.5 or 1.0 for diploid organisms).
	clipped base percentage	Percentage of the bases that are soft-clipped or hand-clipped during mapping.
SNV calling	variant count	Total number of variants in the target region.
	known variants percentage	Percentage of variants that are known polymorphisms in the dbSNP database.
	high-impact variants percentage	Percentage of variants with high impact on the protein, i.e. stop-gain, stop-loss, frameshift, splice-acceptor or splice-donor variants.
	homozygous variants percentage	Percentage of variants that are called as homozygous.
	indel variants percentage	Percentage of variants that are insertions/deletions.
	transition/transversion ratio	Transition/transversion ratio of single nucleotide variants.
CNV calling	CNV count	Number of high-quality CNVs called by ClinCNV. High-quality means that log-likelihood is bigger or equal to 20.

	coverage profile correlation	Mean correlation between the sample coverage profile and the coverage profiles of the referenc
	number of iterations	Number of iterations done by ClinCNV when calling CNVs. CNV calling is repeated with increased log-likelihood threshold as long as too many CNVs are detected. Normally only one iteration is done.
	percentage losses	Percentage of copy-number losses based on high-quality calls on autosomes. High-quality means that log-likelihood is bigger or equal to 20.
	percentage gains	Percentage of copy-number gains based on high-quality calls on autosomes. High-quality means that log-likelihood is bigger or equal to 20.
SV calling	SV count	Number of structural variants called that PASS filters.
	percentage deletions	Percentage of structural variants that PASS filters and are deletions.
	percentage duplications	Percentage of structural variants that PASS filters and are duplications.
	percentage insertions	Percentage of structural variants that PASS filters and are insertions.
	percentage inversions	Percentage of structural variants that PASS filters and are inversions.
	percentage breakends	Percentage of structural variants that PASS filters and are breakends.

Supplementary Table S2: Disease group distribution and report outcome of GS samples

	Exome				Genome			
**Disease groups**	**Total**	**Solved cases**	**Cases with VUS**	**Unsolved cases**	**Total**	**Solved cases**	**Cases with VUS**	**Unsolved cases**
Diseases of the nervous system	**107**	25.23%	28.97%	45.79%	**691**	15.20%	29.38%	55.43%
Mental. behavioural or neurodevelopmental disorders	**522**	28.16%	30.84%	41%	**164**	21.95%	40.24%	37.80%
Neoplasms	**37**	18.92%	2.7%	78.38%	**492**	13.62%	14.02%	72.36%
Diseases of the visual system	**1**	100%	0%	0%	**334**	44.61%	26.95%	28.44%
Endocrine. nutritional or metabolic diseases	**110**	21.82%	15.45%	62.73%	**44**	15.91%	25%	59.09%
Diseases of the musculoskeletal system or connective tissue	**66**	16.67%	39.39%	43.94%	**54**	18.52%	24.07%	57.41%
Diseases of the ear or mastoid process	**0**	0%	0%	0%	**72**	26.39%	45.83%	27.78%
Developmental anomalies	**58**	37.93%	5.17%	56.90%	**12**	25%	16.67%	58.33%
Diseases of the immune system	**60**	13.33%	28.33%	58.33%	**9**	11.11%	22.22%	66.67%
Diseases of the circulatory system	**24**	12.50%	54.17%	33.33%	**33**	9.09%	30.30%	60.61%
Diseases of the genitourinary system	**3**	0%	33.33%	66.67%	**43**	20.93%	16.28%	62.79%
Diseases of the blood or blood-forming organs	**11**	27.27%	36.36%	36.36%	**15**	13.33%	33.33%	53.33%
Other diseases	**17**	41.18%	29.41%	29.41%	**14**	35.71%	21.43%	42.86%
Sum	**1016**	25.59%	27.46%	46.95%	**1977**	21.04%	26%	52.96%

Supplementary Table S3: Ancestry distribution of diagnostic cases from index samples sequenced in 2022

Ancestry	Exome	Genome
EUR	866 (85.24%)	1844 (93.27%)
ADMIXED/UNKNOWN	105 (10.33%)	84 (4.25%)
EAS	4 (0.39%)	12 (0.61%)
AFR	12 (1.18%)	14 (0.71%)
SAS	29 (2.85%)	23 (1.16%)
Total	1016	1977

Supplementary Table S4: List of specific diagnoses solved by ES and GS in 2022

Disease groups	Genes of specific diagnoses solved by ES	Genes of specific diagnoses solved by GS
Diseases of the nervous system	*ABCD1, CACNA1A, CDKL5, COL4A1, CTNS, DAGLA, HTT (RE), IRF2BPL, MFN2, MPZ, MT-TK, NF2, PCDH19, POLG, POLG2, PRRT2, SPAST, SPG11, SPG7, TSC2, WARS2*	*ABCD1, ACTB, ADAR, ADCY5, AFG3L2, AMPD1, ANG, ANO10, AP5Z1, AR (RE), ATP1A3, ATXN2 (RE), ATXN3, BRPF1, C9orf72 (RE), CACNA1A, CNGB1, CYP7B1, FARS2, FLNC, GBE1, GFAP, GJB1, GJC2, GLA, GRN, HADHA, HTRA1, HTT (RE), JAG1, KRIT1, LAMB1, MFN2, MME, MT-ATP6, NEU1, NFKB2, NIPA1, NKX2-1, NOTCH3, OPTN, PAH, PIEZO2, POLR3A, PRKCG, PRNP, PSEN1, PSEN2, REEP1, RORA, SACS, SETX, SLC20A2, SLC2A1, SOD1, SPAST, SPG7, SPG7, STUB1, TARDBP, TBK1, TBP (RE), TTBK2, TUBB3, TWNK, UCHL1,*
Mental. behavioural or neurodevelopmental disorders	*ACAN, ACTB, ADNP, AFG2B, ALDH1A3, ALDH4A1, AMPD1, ANKRD11, ATAD3A, ATP1A2, ATP1A3, BBS2, BCL11A, CAMTA1, CCND2, CHD3, CHD7, CHD8, CNGB3, CNKSR2, CREBBP, CTNNB1, CUL7, DDX3X, EBF3, ECM1, EEF1A2, EHMT1, EHMT1, EIF3F, FANCD2, FBXO11, FGFR3, FOXP1, GRIN2A, GRIN2B, HEXA, HSD17B10, IFIH1, IQSEC2, ITPR1, KAT6B, KCNB1, KCNQ2, KMT2A, KMT2E, KMT5B, LAMB1, MAP1B, MECP2, MED12, MED13, MTFMT, MYT1L, NALCN, NEXMIF, NF1, NFIB, OCA2, ORC6, PAH, PCDHGC4, PHF6, PHKA2, PIGA, POLR3B, POMGNT1, POMT1, UCK1, PPP1CB, PRR12, PTPN11, QRICH1, RAI1, RARS1, RNASET2, RPS6KA3, SCN11A, SCN1A, SELENON, SETD5, SGSH, SHANK1, SHANK3, SLC38A8, SMARCA2, SOX5, SOX9, SRCAP, TCF12, TCF20, TCF4, THRA, TLK2, TNRC6B, TRPS1, TSEN54, TUBA1A, TUBB2A, UBR7, WAC, WDR37, YARS1, ZNF148*	*ABCA12, AP2S1, AUTS2, BBS1, BBS9, BCL11B, CACNA1C, CHST14, COL1A1, COL4A5, FBN1, JARID2, KCND3, KCNJ1, KMT2D, MECP2, MORC2, NR2F1, NRCAM, PCDH12, PLP1, RNU4ATAC, SETD5, TNRC6B, TUBB, FMR1 (RE)*
Neoplasms	*ATM, BRCA1, MUTYH, PTEN, SCN8A, TP53*	*APC, ATM, BARD1, BRCA1, BRCA2, CHEK2, HOXB13, MLH1, MSH2, MSH6, NF1, PALB2, RAD51C, RAD51C, RAD51D, SDHB, TP53, TSC1*
Diseases of the visual system	*USH2A*	*ABCA4, ADGRV1, AIPL1, AP5Z1, ATAD3A, BBS1, BBS1, BBS10, BEST1, BEST1, CDH23, CEP290, CEP78, CERKL, CHM, CLRN1, CNGA3, CNGB3, CYP1B1, ELOVL4, EYS, GUCA1A, KCNV2, MAK, MERTK, MT-ATP6, MT-*

		ND4, MT-ND6, MYO7A, NR2E3, OPA1, OPA3, OTX2, PAX6, PDE6A, PDE6B, PITX3, PMM2, PROM1, PRPF31, PRPF8, PRPH2, PRPH2, RHO, RP1, RP1L1, RP2, RPGR, RPGR, RS1, SPATA7, TMEM126A, TUBB4B, TULP1, USH1C, USH2A, VPS13B, WFS1
Endocrine. nutritional or metabolic diseases	*MT-TL1, NLRP3, RASA1, HBB, ATP7B, SLC2A2, AVPR2, TNFAIP3, ATM, NF1, UGT1A1, BTK, ARSA, SLC19A3, CFTR, ADAR, MT-ATP6, YARS1, PAX8, SLC12A3, BCS1L, SNX14*	*CLCN1, CYP11A1, HFE, LDLR, TG*
Diseases of the musculoskeletal system or connective tissue	*AR (RE), CLCN1, COL2A1, COL6A3, FBN1, HNF1A, TTN, ZC4H2*	*AHCY, ANO5, CBFB, CLCN1, COL2A1, FBN1, HPDL, LMNA, NF1, PHEX*
Diseases of the ear or mastoid process		*GJB2, MT-CO1, MYO6, MYO7A, PTPRQ, SLC26A4, STRC, TECTA, TMIE*
Developmental anomalies	*ANK1, CEP290, CHD7, COL1A1, GBE1, GREB1L, IRF6, KMT2D, LPAR6, RB1, MYRF, NIPBL, PIEZO1, WNT10A*	*CHST14, LDLR, RBM8A*
Diseases of the immune system	*CYBB, ELANE, MEFV, NLRP3, RAG1*	*BTK*
Diseases of the circulatory system	*MYBPC3, CHD7, KCNQ1*	*DSP, TPM1, TTN*
Diseases of the genitourinary system		*AR, HSD17B3, SRD5A2, UMOD*
Diseases of the blood or blood-forming organs	*ABCC6, ADAMTS13, CFTR*	*ENG, MYH9*
Other diseases	*ACTG2, CCDC103, NF1, PLG*	*KRIT1, NF1, NLRP3*

Genes affected by disease-causal small variants and repeat expansions (RE) sorted by disease groups.

Supplementary Table S5: Repeat motives/regions screened with Expansion Hunter tool

Chromosome	Position Start	Position End	Gene	Repeat unit
chr1	149390802	149390841	*NOTCH2NL*	GGC
chr2	190880872	190880920	*GLS*	GCA
chr3	63912684	63912714	*ATXN7*	GCA
chr3	63912714	63912726	*ATXN7*	GCC
chr3	129172576	129172656	*CNBP*	CAGG
chr3	129172656	129172696	*CNBP*	CAGA
chr3	129172696	129172732	*CNBP*	CA
chr4	3074876	3074933	*HTT*	CAG
chr4	3074939	3074966	*HTT*	CCG
chr4	39348424	39348479	*RFC1*	AARRG
chr4	41745972	41746032	*PHOX2B*	GCN
chr5	146878727	146878757	*PPP2R2B*	GCT
chr6	16327633	16327723	*ATXN1*	TGC
chr6	170561906	170562017	*TBP*	GCA
chr9	27573528	27573546	*C9ORF72*	GGCCCC
chr9	69037261	69037286	*FXN*	A
chr9	69037286	69037304	*FXN*	GAA
chr11	119206289	119206322	*CBL*	CGG
chr12	6936716	6936773	*ATN1*	CAG
chr12	50505001	50505022	*DIP2B*	GGC
chr12	111598949	111599018	*ATXN2*	GCT
chr13	70139353	70139383	*ATXN8OS*	CTA
chr13	70139383	70139428	*ATXN8OS*	CTG
chr13	102161576	102161726	*FGF14*	GAA
chr14	23321472	23321490	*PABPN1*	GCG
chr14	92071009	92071042	*ATXN3*	GCT
chr15	22786677	22786701	*NIPA1*	GCG
chr16	87604287	87604329	*JPH3*	CTG
chr18	55586155	55586227	*TCF4*	CAG
chr19	13207858	13207897	*CACNA1A*	CTG
chr19	14496041	14496074	*GIPC1*	CCG
chr19	45770204	45770264	*DMPK*	CAG
chr20	2652733	2652757	*NOP56*	GGCCTG

chr20	2652757	2652775	*NOP56*	CGCCTG
chr21	43776443	43776479	*CSTB*	CGCGGGGCGGGG
chr22	45795354	45795424	*ATXN10*	ATTCT
chrX	67545316	67545385	*AR*	GCA
chrX	147912050	147912110	*FMR1*	CGG
chrX	148500631	148500691	*AFF2*	GCC

Supplementary Figure S1: Age distribution of index samples in 2022

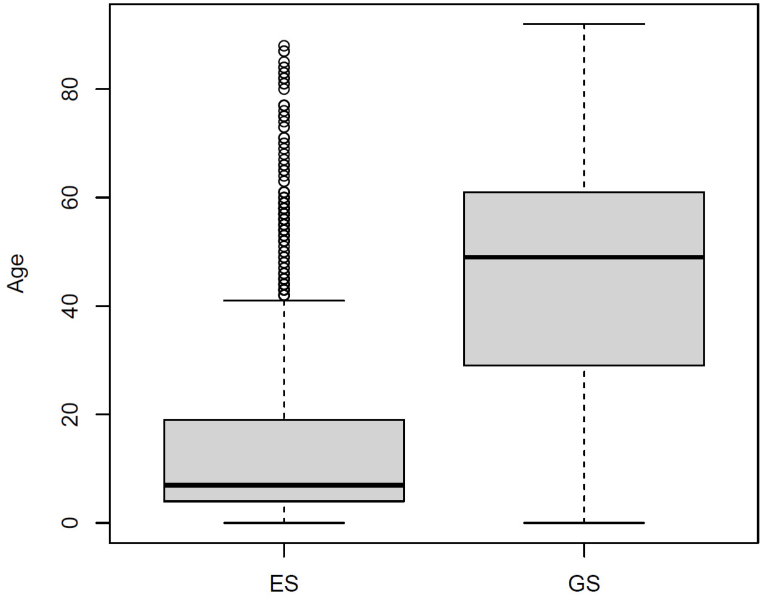

Supplement: Supplementary file 1 [file genes-15-00136-s001.zip › genes-2809987-supplementary.pdf]
